# Piezoresistivity of InAsP Nanowires: Role of Crystal Phases and Phosphorus Atoms in Strain-Induced Channel Conductances

**DOI:** 10.3390/molecules24183249

**Published:** 2019-09-06

**Authors:** In Kim, Han Seul Kim, Hoon Ryu

**Affiliations:** National Institute of Supercomputing and Networking, Korea Institute of Science and Technology Information, Daejeon 34141, Korea (I.K.) (H.S.K.)

**Keywords:** piezoresistivity, indium-arsenide-phosphide (InAsP) nanowires, electronic structure simulations, density functional theory

## Abstract

Strong piezoresistivity of InAsP nanowires is rationalized with atomistic simulations coupled to Density Functional Theory. With a focal interest in the case of the As(75%)-P(25%) alloy, the role of crystal phases and phosphorus atoms in strain-driven carrier conductance is discussed with a direct comparison to nanowires of a single crystal phase and a binary (InAs) alloy. Our analysis of electronic structures presents solid evidences that the strong electron conductance and its sensitivity to external tensile stress are due to the phosphorous atoms in a Wurtzite phase, and the effect of a Zincblende phase is not remarkable. With several solid connections to recent experimental studies, this work can serve as a sound framework for understanding of the unique piezoresistive characteristics of InAsP nanowires.

## 1. Introduction

Piezoelectric and piezoresistive effects, the capability of converting mechanical stress to electrical signals, induced much intention as a novel candidate of nanoscale sensors for detection of small forces or pressures [1,2,3,4] owing to their special characteristics that enable uncomplicated device designs with relatively low power consumption in operations [5]. Among III-V semiconductor devices, nanowires particularly have obtained widespread interests as they generally have high mobility and direct band gap [6,7,8,9,10,11]. Additionally, III-V nanowires based on GaN [12], InAs [13,14], and InGaAs [15], show remarkable piezoresistive behaviors driven by tensile stress.

Recently, it has been found that InAsP nanowires, which have two crystal phase of a Wurtzite (WZ) and Zincblende (ZB), also possess piezoresistivity [16]. A following theory theoretically predicted this phenomenon may be due to the increase of mobility coming from strain-induced reduction of band gap energies in nanowires of a WZ phase [17]. The prediction is, however, deduced only with investigation of a single WZ configuration so its comparison to a ZB phase is not clear. Another theory work on InAs systems reported the enhancement of carrier transport is mainly due to a WZ phase [18]. But, the origin of enhanced conductivity is still unclear in viewpoints of crystal phases; only suspected by the effects of phonon scattering, and the contribution of the two crystal phases is ambiguous since the work considers supercells that contain both phases. Moreover, the effects of tensile stress are not addressed. As far as we know, no study so far has presented in-depth discussion on the effects of strain in different crystal phases and the contribution of P atoms to the piezoresistivity in InAsP nanowires.

Here we explore the piezoresistivity of InAsP nanowires using Density Functional Theory (DFT) simulations in a systematic manner. We model various configurations of an InAsP crystal according to doping positions in nanowires of a WZ and ZB phase, apply uniaxial tensile strain along the direction of carrier transport, and study corresponding electronic structures and conducting behaviors to address the role of crystal phase and tensile stress on the piezoresistivity of nanowires. The contribution of P atoms in nanowires of different phases is also uncovered, which may serve as a practical guideline for potential device designs.

## 2. Methods

**Figure 1:**
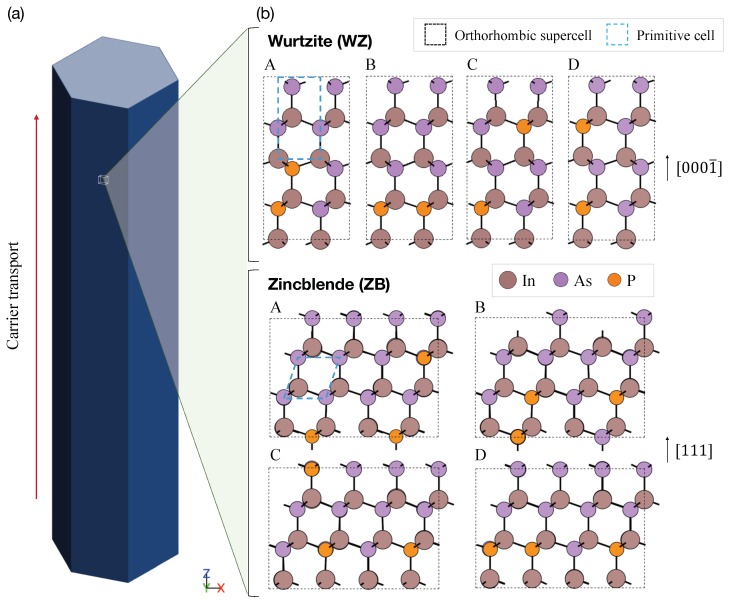
**Target structures for electronic structure simulations.** (**a**) Schematic of InAsP nanowires. The red arrow indicates the direction of electron transport. (**b**) The ball-and-stick model of InAs${}_{0.75}$P${}_{0.25}$ configurations considered in this work: a Wurtzite (WZ) and Zincblende (ZB) phase. Dashed black and blue lines show the boundary of simulated orthorhombic supercells and primitive cells, respectively. Brown, pink, and orange balls represent In, As and P atoms, respectively. Transport directions InAsP nanowires in a WZ and ZB phase are $\left[000\bar{1}\right]$ and [111], respectively, and are aligned to the *z* axis. Atomic configurations are visualized with ASE package [30].

In order to obtain optimized atomic structures of InAsP nanowires in a WZ and ZB phase, we adopt the projector-augmented wave (PAW) method that is implemented in the Vienna Ab-initio Simulation Package (VASP) [19]. A set of the 4 supercell configurations is considered per each crystal phase to include the effects of random placements of P atoms, as shown in Figure 1. For all the electronic structure simulations, we adopted the flavor of Perdew-Berke-Ernzerhof generalized gradient approximation revised for solids (PBEsol) [20]. A set of plane wave basis is chosen with a 450 eV energy cut-off. The convergence criterion of self-consistent field is set to $10^{-7}$ eV with a Γ-centered 16×16×16 *k*-points mesh with no origin shift. Geometry optimization of supercells is conducted with a convergence criterion of $10^{-6}$ eV/Å. Based on this result of atomic relaxation, electronic structures are computed with the SIESTA code that employs numerical atomic orbital (NAO) basis [21]. PBEsol is utilized for the exchange-correlation (XC) term, and the atomic cores are replaced with Troullier-Martins Norm-conserving pseudopotentials [22]. Split-valence double-ζ basis with a polarization orbitals (DZP) with 0.1 eV energy shift [23] is chosen for all the atomic species. Integrals of self-consistent terms in Kohn-Sham Hamiltonian are obtained with aids of a real-space grid of 300 Ry cutoff where the electron density is projected. A set of 20×20×20 *k*-point grids is employed to sample the 1st Brillouin zone.

Electron mobility is evaluated with the 2nd derivatives of sub-bands, assuming the E-*k* relationship of sub-bands is parabolic in the vicinity of conduction and valence band edges [24]. In order to predict the conductance properties from band structures, we adopted the Boltzmann transport equation, where the energy-projected conductivity tensors are defined as:

(1) $$\sigma_{\alpha \beta }\left(\epsilon ,T\right)=\int \sum_{b}v_{b,k}⊗v_{b,k}\tau_{b,k}\delta \left(\epsilon -\epsilon_{b,k}\right)\frac{dk}{8\pi^{3}}$$

where ϵ,*T*,τ represent the energy, temperature, mean-free time. The subscript *b* runs over sub-bands for α,β=x,y,z (Figure 1a), and $v_{b}\left(i,k\right)=\left(1/\hbar \right)\left(\partial \epsilon_{i,k}/\partial k_{b}\right)$ is the group velocity of electrons near sub-band edges. From Equation (1), the conductivity tensors $\sigma_{\alpha \beta }\left(T,\mu \right)$ can be expressed as

(2) $$\sigma_{\alpha \beta }\left(\mu ;T\right)=\frac{1}{\Omega }\int \sigma_{\alpha \beta }\left(\epsilon \right)\left[-\frac{\partial f_{\mu }\left(T,\epsilon \right)}{\partial \epsilon }\right]d\epsilon$$

where Ω is the volume of a target supercell and $f_{\mu }\left(\epsilon \right)$ is the Fermi-Dirac distribution function. BoltzTraP2 code [25], which has been widely used in studying resistivity of LaNiO${}_{3}$ thin film [26], Seebeck coefficient of CoSi [27], and charge transport of CH${}_{3}$NH${}_{3}$PbI${}_{3}$ perovskite structures [28], is employed to evaluate the electron conductivity $\sigma_{\alpha \beta }\left(T,\mu \right)$ from band structures obtained with DFT simulations. The total time needed to solve the electronic structure of a single WZ and ZB supercell is approximately 8 and 34 h on average with two 20-core 2.4 GHz Intel Xeon 6148 processors in the NURION high performance computing resource [29], respectively.

## 3. Results & Discussion

As a target composition of InAs${}_{x}$P${}_{1-x}$ alloys, we focus on x=0.75 analogously to the previous experimental study of InAsP nanowires [16]. Orthorhombic supercells in a WZ and ZB phase are constructed with the primitive cell of each crystal phase by defining the lattice by $\left(a,b,c\right)=\left(\sqrt{3}a_{0},a_{0},c_{0}\right)$ and $\left(\sqrt{6}a_{0},\sqrt{2}a_{0},\sqrt{3}a_{0}\right)$ where $a_{0},c_{0}$ are the cubic lattice constants for WZ and ZB, respectively [31]. The WB and ZB supercells consist of 16 and 48 atoms for WZ and ZB, respectively. The direction of carrier transport is set to the *z* axis (Figure 1b), which corresponds to $\left[000\bar{1}\right]$ and [111] for WZ and ZB nanowires, respectively. The average values of lattice constants along (x,y,z) direction are noted as (a,b,c), and the constant values for optimized orthorhombic structures are presented in Table 1. We note that the optimized lattice parameters are in good agreement with the ones obtained theoretically and experimentally for InAs and InP structures [32,33,34,35,36] with a consideration of Vegard’s law [37].

**Figure 2:**
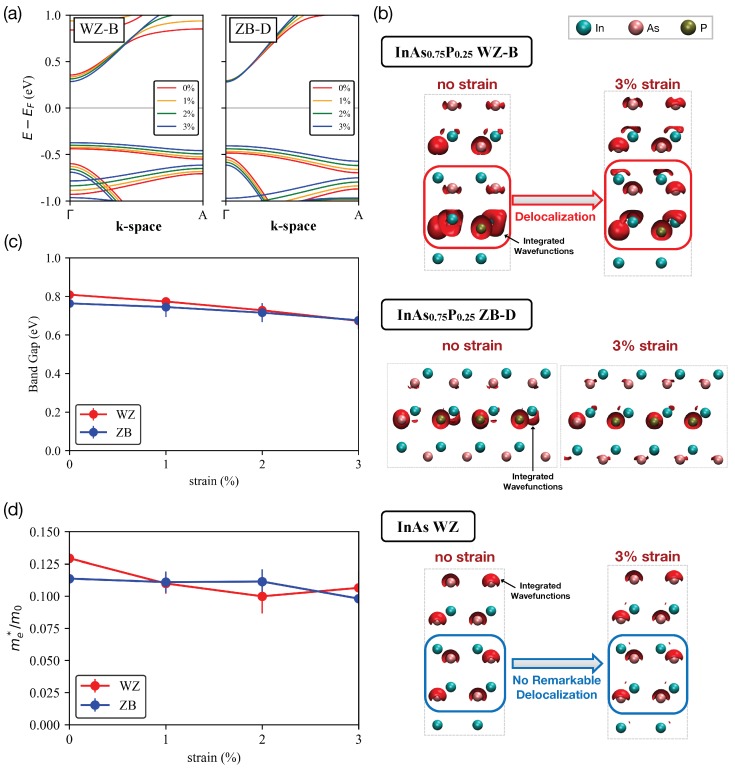
**Change of nanowire properties with respect to tensile stress.** (**a**) Band structures of the WZ-B and ZB-D supercell along the transport direction shows that the band gap of the WZ-B nanowire clearly reduces with increased strain, while this pattern is not clearly observed in the ZB-D case. (**b**) Isosurfaces of the local density of states (LDOS) integrated near the conduction band edge of InAs WZ, InAs${}_{0.75}$P${}_{0.25}$ WZ-B, and InAs${}_{0.75}$P${}_{0.25}$ ZB-D nanowire at a 0% and 3% stress (visualized with VMD [40]). Cyan, pink, and gold balls represent In, As, and P atoms, respectively. In the InAs${}_{0.75}$P${}_{0.25}$ WZ-B nanowire, increased tensile stress clearly delocalizes carriers over adjacent stacking layers, while the strain-driven delocalization is not remarkable in InAs WZ and InAs${}_{0.75}$P${}_{0.25}$ ZB-D nanowires. (**c**) Band gap energies and (**d**) effective masses of the lowest conduction subbands of InAsP nanowires that are averaged against the 4 supercells (A-D) shown in Figure 1b. In general, effective masses and band gap energies of WZ nanowires are more sensitive to those of ZB nanowires, and both quantities reduce with increased tensile stress (up to a 2% stress for effective masses).

To see the effect of tensile stress on nanowire electronic structures, we applied uniaxial tensile stress along the *z*-direction by gradually increasing the *c* value (Table 1) by 3% while keeping the unit cell volume unchanged. In Figure 2a, we plot band structures of the WZ-B and ZB-D supercells (see Figure 1b) as a function of tensile stress. Here, it is clear that the conduction band minima (CBM) in a WZ phase are more sensitive to the stress than those of a ZB phase is, so their energetic positions approach to the Fermi energy ($E_{F}$) more noticeably with increased stress. Under the *z*-directional uniaxial strain, the xy plane experiences a compressive radial strain [39], so the In-P bonding length projected onto a xy plane is reduced. Consequently, the stability of In-P bonds increases, which can be one of the reasons for decrease of CBM. Figure 2b shows the isosurfaces of the local density of states (LDOS) that are integrated in an energy window of from $E_{F}$ to 0.1 eV above CBM, and are overlaid on top of atomic structures of InAs WZ, InAs${}_{0.75}$P${}_{0.25}$ WZ-B and ZB-D sample. In the WZ-B case, increased tensile stress delocalizes electrons near group III atoms (a red box with a “Delocalization” label in top op Figure 2b) such that they can talk to electrons in the next atomic layers along the *z*-direction. In the ZB-D sample(middle of Figure 2b), however, such delocalization is not as strong as the one found in the WZ-B sample. So, we claim the higher conductivity of WZ nanowires originates from the stronger carrier delocalization. Compared to the result of InAs WZ samples (bottom of Figure 2b), the contribution of P atoms to electron conductance is evident, as increased strain does not necessarily delocalize electrons with no P atoms.

With no strain, the average of band gap energies of WZ and ZB nanowires are 0.809 and 0.764 eV, respectively, as shown in Figure 2c, and these values are comparable to the ones reported for InAs${}_{0.6}$P${}_{0.4}$ and InAs${}_{0.8}$P${}_{0.2}$ samples [41], and a bit larger than the band gap of pure InAs (∼0.35 eV) [15]. Strain-free WZ samples exhibit slightly larger band gap energy than ZB samples similarly as the case of InAs [42,43,44]. InAs${}_{0.75}$P${}_{0.25}$ nanowires in a WZ phase may thus possess a lower electron conductivity than those in a ZB phase at a strain-free condition. With a 3% strain, however, the average band gap of WZ and ZB samples are reduced by 0.14 and 0.088 eV, respectively. The faster reduction of band gap energies with increased tensile stress should be one of the factors contributing to the rapid increase of the electron population, particularly compared to the case of ZB nanowires. While the band gap reduction of InAsP WZ nanowires (0.14 eV at a 3% stress) turns out to be similar to the value (0.15 eV at a 3% stress) reported for the InAs WZ case [15,33], the stronger delocalization of carriers (Figure 2b) can make InAsP nanowires as a better conductor under tensile stress.

Increased strain reduces curvatures (effective masses) of the lowest a few conduction sub-bands, and this reduction is faster in a WZ than a ZB phase as shown in Figure 2d, the average strain-free effective mass of the lowest conduction sub-band becomes 0.129 and 0.114 of the free-electron mass in WZ and ZB nanowires, respectively. As the strain applied to WZ structures, the mass decreases to 0.110, 0.100, and then increased to 0.106 at a 1, 2, and 3% strain, respectively. In the case of ZB, the mass does not change (∼0.11) up to a ∼2% tensor and reduces to 0.098 at 3%. This reveals that the effective mass in a WZ phase is more sensitive to tensile strain than that in a ZB phase is. It may be thus safe to conclude that electrons in strained WZ InAs${}_{0.75}$P${}_{0.25}$ nanowires (up to ∼2%) move faster along the transport direction than in ZB ones at the same condition in terms of the strain magnitude. At a 3% stress, electrons may move slower in a WZ phase than in a ZB phase due to their larger effective masses. This however would not necessarily indicate that the conductivity of WZ nanowires is lower than that of ZB nanowires, because the LDOS (particularly the carrier delocalization we discuss in Figure 2b) profile will also affect the conductivity.

**Figure 3:**
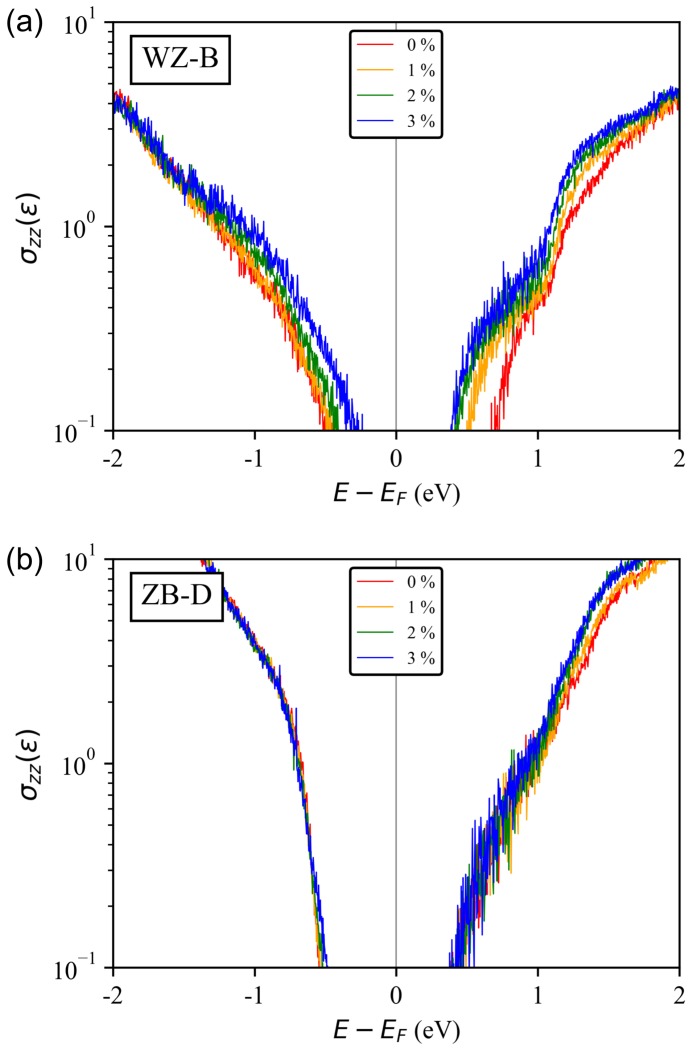
**Energy-projected conductivity tensor ($\sigma_{zz}\left(\epsilon \right)$) at T = 300 K, calculated for (a) InAs${}_{0.75}$P${}_{0.25}$ WZ-B and (b) ZB-D nanowires.** In the WZ-B nanowire, we observe the remarkable increase of $\sigma_{zz}\left(\epsilon \right)$ near $E_{F}$ with increasing uniaxial strain, but no such changes are found near valence and conduction band edge in the ZB-D structure.

To find another correlation between strain and electron transport, we compute the gradient of a symmetry-adapted Fourier interpolation of sub-band energies, and obtain the profile of energy-dependent carrier conductivity along the transport (*z* in Figure 1b) direction with Equation (1) ($\sigma_{zz}$) at T = 300 K. Figure 3 shows $\sigma_{zz}\left(\epsilon \right)$ of a WZ-B and ZB-D nanowire. Here, we focus on the $\sigma_{zz}\left(\epsilon \right)$ profile near the energy range above $E_{F}$ because it then could present a sound clue for electron conductivity when the channel is populated with positive gate biases that will shift down the $\sigma_{zz}\left(\epsilon \right)$ profile towards $E_{F}$ (the shape of $\sigma_{zz}\left(\epsilon \right)$ distribution even does not change if the self-consistency is ignored). Figure 3 indicates, in the energy range of $\left[E_{F},E_{F}+1\;\mathrm{eV}\right]$, there is a remarkable increase of $\sigma_{zz}\left(\epsilon \right)$ in a WZ phase with increasing strain, while this increase is not quite clear in a ZB phase. In all the structures in each crystal phase (A-D in Figure 1b), the pattern of $\sigma_{zz}\left(\epsilon \right)$ turns out to be quite similar as what is shown in Figure 3. Also, we note that the result in Figure 3 is consistent with the aforementioned observations in that the property of WB InAsP nanowires changes to the direction of enhancing conductivity as stress increases, i.e., decrease of band gap energies, delocalization and reduced effective masses of electrons.

**Figure 4:**
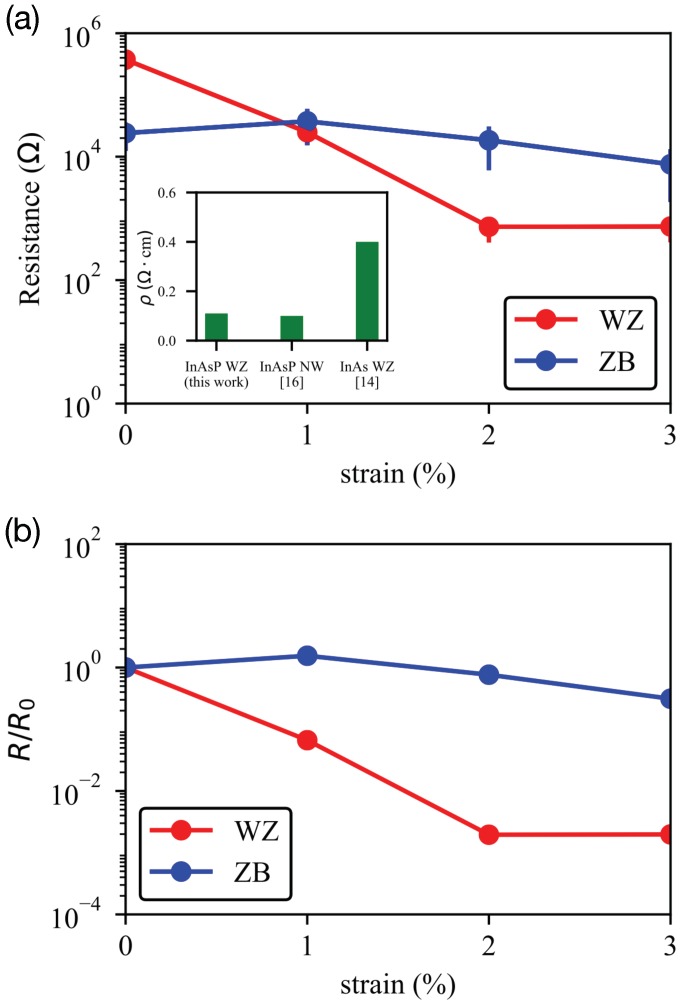
**Sensitivity of channel resistance of InAs${}_{0.75}$P${}_{0.25}$ WZ and ZB nanowires to tensile stress.** (**a**) Resistance along transport direction, the value averaged against the four supercells, is shown as a function of tensile stress. The inset shows the resistivity calculated in this work and experimental results [14,16]. (**b**) Resistances (*R*) scaled to their strain-free values ($R_{0}$). With a 3% strain, the resistance of InAsP WZ nanowires reduces by a factor of $10^{3}$, while this factor becomes much smaller (around $10^{-1}$) in ZB nanowires.

The resistance *R* along transport directions of supercells can be calculated with conductivities that can be evaluated by integrating the energy-dependent conductivity profiles (Equations (1) and (2)), lattice parameters (Table 1), and the Ohm’s law R=ρl/*A* [7], where ρ is the resistivity (the inverse of conductivity), and (l,A) are the length and cross-section area of the orthorhombic cell. Figure 4 shows the R value (averaged for the 4 supercells in each phase) as a function of the strain magnitude. In WZ nanowires, we see that increased stress reduces R, and this pattern is much more obvious than the one observed in ZB nanowires (Figure 4a). The *R* and ρ of WZ cases at zero-strain are $3.7\times 10^{5}$ Ω and 0.11 Ω·cm in average, respectively. Here we note the simulation results are well connected to the experimental work reported by Lee et al. [16], which observed the ρ of an InAs${}_{0.75}$P${}_{0.25}$ nanowire to be around 0.1 Ω·cm under no stress. It should be also noted that this value is smaller to the experimental result (0.4 Ω·cm) reported for a WZ InAs nanowire by Zheng et al. [14], presenting a sound theoretical clue for the contribution of P dopants to carrier conductivities. As the stress increases, the resistance of WZ nanowires dramatically reduces. Its relative reduction, represented with *R*/$R_{0}$ ($R_{0}$ is the strain-free resistance) in Figure 4b, thus reaches to around $10^{-3}$ at a 3% stress. On the other hand, the strain-driven reduction of *R* and ρ in ZB nanowires is not as remarkable as what we see from WB nanowires, so the relative reduction of channel resistance is <$10^{-1}$ at a 3% stress.

## 4. Conclusions

In-depth analysis on piezoresistive behaviors of InAsP nanowires is conducted with aids of simulations based on Density Functional Theory. InAsP nanowires in a Wurtzite (WZ) and Zincblende (ZB) phase, and InAs nanowires are employed as targets of modeling, and discussion on InAsP nanowires are focused on P = 25% alloys for connections to latest experimental studies. The huge reduction of channel resistivity driven by external uniaxial tensile stress, which is uniquely observed in InAsP nanowires of a WZ crystal phase, is theoretically confirmed to be due to the reduction of band gap energies and the increased effective masses of sub-bands near conduction band minimum. Phosphorous atoms play a critical role in the strain-driven enhancement of conductance, invoking delocalization of carriers among inter-atomic layers along the transport direction, and this phenomenon is uniquely observed only in WZ phase. With the findings in overall, we may conclude that the most decisive factor for the strong piezoresistivity of InAsP nanowires should be their crystal phase. Our findings of the remarkable enhancement of conductance of InAsP nanowires by uniaxial stretching give insight on the efficient designs of piezoresistive sensors through strain engineering.

## Figures and Tables

**Table 1:** Geometry-optimized lattice parameters (a,b,c) of InAs, InAs${}_{0.75}$P${}_{0.25}$, and InP Wurtzite (WZ) and Zincblende (ZB) structures (units in Å) used in this study. Lattice parameters of orthorhombic supercells are defined by $\left(a,b,c\right)=\left(\sqrt{3}a_{0},a_{0},c_{0}\right)$ and $\left(\sqrt{6}a_{0},\sqrt{2}a_{0},\sqrt{3}a_{0}\right)$ where $a_{0},c_{0}$ are the cubic lattice constants in a WZ and ZB phase, respectively. The values in parentheses are the percentage error of the model compared to experimental values. Note that the values of InAs${}_{0.75}$P${}_{0.25}$ are averaged against the 4 supercells that are employed to consider the random dopant placements (see Figure 1b).

WZ	Orthorhombic (Error %)			Expt.		
	*a*	*b*	*c*	$a_{0}$	$c_{0}$	Ref.
InAs	7.4035 (0.01)	4.2744 (0.01)	7.0257 (0.01)	4.2742	7.0250	[38]
InP	7.1822 (0.10)	4.1466 (0.10)	6.8098 (0.13)	4.1423	6.8013	[34]
InAs${}_{0.75}$P${}_{0.25}$	7.3630	4.2503	13.9689			
**ZB**	a	b	c	$a_{0}$		**Ref.**
InAs	14.8456 (0.28)	8.5711 (0.28)	10.4975 (0.28)	6.044		[36]
InP	14.3931 (0.34)	8.3098 (0.34)	10.1774 (0.34)	5.856		[36]
InAs${}_{0.75}$P${}_{0.25}$	14.7286	8.5028	10.4155			
